# Sex-Inclined Piwi-Interacting RNAs in Serum Exosomes for Sex Determination in the Greater Amberjack (*Seriola dumerili*)

**DOI:** 10.3390/ijms24043438

**Published:** 2023-02-08

**Authors:** Qiuxia Deng, Na Zhao, Xiaoying Ru, Ruijuan Hao, Bo Zhang, Chunhua Zhu

**Affiliations:** 1 Fisheries College, Guangdong Ocean University, Zhanjiang 524088, China; 2 Southern Marine Science and Engineering Guangdong Laboratory (Zhanjiang), Zhanjiang 524025, China; 3 Guangdong Research Center on Reproductive Control and Breeding Technology of Indigenous Valuable Fish Species, Zhanjiang 524088, China

**Keywords:** piwi-interacting RNAs, serum exosome, *Seriola dumerili*, sex inclination, molecular markers

## Abstract

The greater amberjack (*Seriola dumerili*) is a gonochoristic fish with no sexual dimorphism in appearance, making sex identification difficult. Piwi-interacting RNAs (piRNAs) function in transposon silencing and gametogenesis and are involved in various physiological processes, including sex development and differentiation. Exosomal piRNAs can be indicators for the determination of sex and physiological status. In this study, four piRNAs were differentially expressed in both serum exosomes and gonads between male and female greater amberjack. Three piRNAs (piR-dre-32793, piR-dre-5797, and piR-dre-73318) were significantly up-regulated and piR-dre-332 was significantly down-regulated in serum exosomes and gonads of male fish, compared to female fish, consistent with the serum exosomal results. According to the relative expression of four marker piRNAs derived from the serum exosomes of greater amberjack, the highest relative expression of piR-dre-32793, piR-dre-5797, and piR-dre-73318 in seven female fish and that of piR-dre-332 in seven male fish can be used as the standard for sex determination. The method of sex identification can ascertain the sex of greater amberjack by blood collection from the living body, without sacrificing fish. The four piRNAs did not show sex-inclined expression in the hypothalamus, pituitary, heart, liver, intestine, and muscle tissue. A piRNA–target interaction network involving 32 piRNA-mRNA pairs was generated. Sex-related target genes were enriched in sex-related pathways, including oocyte meiosis, transforming growth factor-beta signaling pathway, progesterone-mediated oocyte maturation, and gonadotropin releasing hormone signaling pathway. These results provide a basis for sex determination in greater amberjack and improve our understanding of the mechanisms underlying sex development and differentiation in the species.

## 1. Introduction

The greater amberjack (*Seriola dumerili*) is economically valuable worldwide and mainly inhabits the deep sea environment, and is characterized by fast growth, good taste, and high nutritional value [1,2,3,4]. Owing to its high economic value, extensive studies have focused on the artificial domestication and breeding of greater amberjack since the 1990s [5]. However, it takes about 3 to 4 years for wild greater amberjack to reach gonadal maturity [6]. Furthermore, due to the lack of a comprehensive understanding of the regularity of gonadal development, greater amberjack broodstocks usually have reproductive disorders under artificial breeding conditions. In addition, greater amberjack is a gonochoristic fish with no sexual dimorphism in appearance [1,7], presenting a challenge in the determination of sex. These factors have greatly limited the development of artificial breeding and large-scale culturing of greater amberjack. Reproductive disorders can be overcome, to a certain extent, by the injection of exogenous reproductive hormones [3,8,9,10,11,12,13,14,15,16,17]. Methods used for sex determination in greater amberjack have various limitations. For example, observations of the gonads (only at the stage of sexual maturity) and histological analyses cause fish damage or even death [18]. The estimation of the 11-ketotestosterone (11-KT) concentration and 11-KT/estradiol (E_2_) ratio in the serum is not effective for young adults with very low blood hormone levels [2]. Sex-associated single nucleotide polymorphisms (SNPs) in greater amberjack are mainly found on chromosome 12 (approximately 7.1 Mbp) [1,19]. However, although specific SNPs are important for artificial breeding and large-scale breeding, sex-specific SNPs for sex determination have not been established in greater amberjack.

Exosomes are 30–150 nm extracellular vesicles with a lipid bilayer structure and contain various nucleic acids, proteins, and lipids [20,21,22]. Exosomes are transported from donor cells to recipient cells and considered important regulatory molecules involved in various functions, including intercellular communication and transport, providing potential biomarkers for diagnosis and monitoring [20,21,22,23,24,25]. Piwi-interacting RNAs (piRNAs) are a class of 26–31 nt small RNAs. They were initially discovered in mammalian germ cells in 2006 [26,27] and are important components of exosomes in teleosts [20]. They have roles in transposon silencing, gametogenesis, the maintenance of genomic integrity, and stem cell lineages [20,28,29,30]. Sex-inclined piRNAs are involved in various physiological processes, including sex development, sex differentiation, sex determination, and sex regulation in aquatic animals [20,30,31,32,33,34]. We have previously shown that sex-inclined exosomal piRNAs could be employed as biomarkers to identify the male and pseudo-male fish, with the same karyotype as female fish and the same physiological characteristics as male fish [34], in half smooth tongue sole (*Cynoglossus semilaevis*) [34,35]. In this study, piRNA profiles in serum exosomes from greater amberjack were characterized to screen differentially expressed piRNAs between sexes. The signature piRNAs and their sex-related targets will guide further studies of the mechanism underlying sex differentiation in greater amberjack.

## 2. Results

### 2.1. Fish Size and Gonad Histology

**Figure 1:**
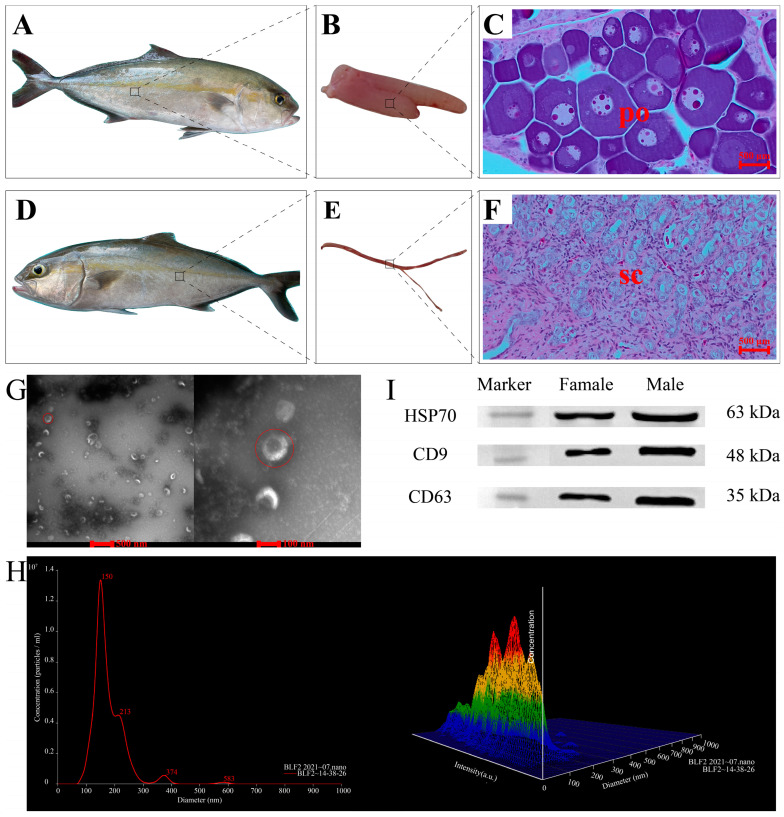
Morphology of greater amberjack and its gonads, histological sections of the gonads, and isolation and identification of exosomes from greater amberjack serum. Morphological and histological results for (**A**–**C**) female greater amberjack; (**D**–**F**) male greater amberjack. (**G**) Electron microscope images of exosomes. The red circle on the left shows the electron microscope image of a exosome at a resolution of 100 nm, while the red circle on the right shows that of the exosome at a resolution of 500 nm. (**H**) Particle size distributions and concentration of exosomes in a female greater amberjack determined by NTA 2.3. **Left**: Line chart. The *x*-axis represents the diameter of the particles, and the *y*-axis represents the concentration of the particles. The red number represents the value corresponding to each peak. **Right**: Three-dimensional graph. The *x*-axis represents the diameter of the particles, and the *y*-axis represents the intensity of the particles, and the *z*-axis represents the concentration of the particles. The color represents the value of the diameter, intensity and concentration of a particle. A redder color indicates a larger value, followed by yellow and green, while a bluer color indicates a smaller value. (**I**) Western blotting for the detection of heat shock protein 70, tetraspanin CD9, and tetraspanin CD63. po, primary oocytes. sc, spermatocytes.

There were no significant differences in body length or weight between the two groups in greater amberjack (male group: 47.33 ± 0.94 cm, 1566.67 ± 94.28 g; female group: 49.33 ± 1.25 cm, 1716.67 ± 164.99 g; independent samples *t*-test, *p* > 0.05; Figure 1A,D). Compared with the testis size, the ovary was significantly larger in greater amberjack (Figure 1B,E). Based on histological sections of gonads of greater amberjack, a typical testicular structure with spermatocytes (sc) was observed in the testis, and the ovarian structure with ovarian lamellae and many primary oocytes (po) was clearly seen in the ovary (500 μm, Figure 1C,F).

### 2.2. Identification and Characterization of Exosomes

High concentrations of exosomes were detected in the serum of greater amberjack. Exosomes were observed as lipid-bilayer nanoscale vesicles (30–150 nm in diameter) with a tea-tray-like structure by transmission electron microscopy (TEM) (Figure 1G). The nanoparticle tracking analysis (NTA) revealed that the mean size and main peaks of exosomal samples were 175.9 nm and 146.9 nm, respectively, and the mean concentration was (9.44 ± 0.253) × 10^8^ particles/mL, in which particles with diameters of 30–100 nm accounted for 37.72% of all particles (Figure 1H). As determined by western blotting (WB), three exosomal markers (heat shock protein 70 (HSP70), tetraspanin CD9, and tetraspanin CD63) were positive in all samples (Figure 1I and Figure S1). The molecular weights of the three exosome markers were approximately 63 kDa (HSP70), 48 kDa (CD9), and 35 kDa (CD63), respectively.

### 2.3. Piwi-Interacting RNAs Profiles in Male and Female Serum Exosomes

**Figure 2:**
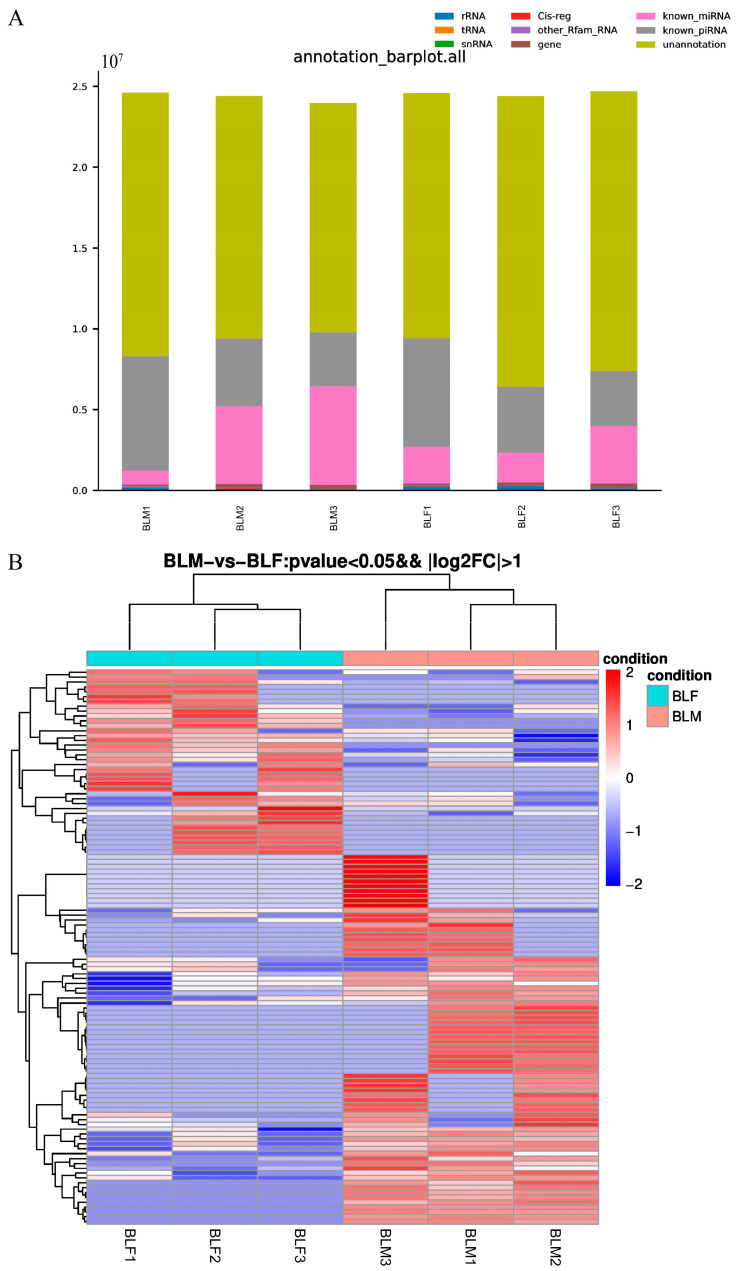
The classification and annotation of all reads and differentially expressed piwi-interacting RNAs from small RNA sequencing results. (**A**) Bar plot of the classification and annotation of all reads in the male and female groups. The *x*-axis represents the sample, and the *y*-axis represents the number of reads of various small RNAs. Annotated non-coding RNAs included ribosomal RNAs, transfer RNAs, small nuclear RNAs, Cis-reg, other Rfam RNA, gene, microRNAs, and piwi-interacting RNAs. (**B**) Hierarchical cluster analysis of 114 differentially expressed piwi-interacting RNAs between the male and female group based on transcripts per million values. The thresholds for the identification of differentially expressed piwi-interacting RNAs were *p* < 0.05 and |log_2_ fold change| > 2.

Total RNAs were isolated from serum exosomes of three male and three female greater amberjacks and used for small RNA sequencing. Raw data have been uploaded to the NCBI website (Accession No. PRJNA896259). The numbers of clean reads (adapter-removed and filtered high-quality reads) in the male group and female group were 23.98–24.62 M and 24.41–24.69 M, respectively. These clean reads were aligned with the Rfam database, GenBank (http://www.ncbi.nlm.nih.gov/genbank/, accessed on 23 November 2021) databases, miRBase database, and piRBase database, and annotated as various non-coding RNAs, including ribosomal RNAs (rRNAs), transfer RNAs (tRNAs), small nuclear RNAs (snRNAs), Cis-reg, other Rfam RNA, gene, microRNAs (miRNAs), and piRNAs (Figure 2A). There were 5291–7374 unique known piRNA-aligned reads in the male group, accounting for 0.98–1.13% of the unique clean reads in respective samples, while there were 5192–7078 in the female group, accounting for 0.90–1.05% of the unique clean reads in respective samples. The numbers of detected known piRNAs in the male group were 1680, 1600, and 1335, respectively, compared with 1256, 1670, and 1211 in the female group.

### 2.4. Screening Differentially Expressed Piwi-Interacting RNAs between Male and Female Greater Amberjack and Target Prediction

Based on TPM, 114 differentially expressed piRNAs were identified between the male group and female group with *p* < 0.05 and |log_2_ fold change (FC)| > 2, of which 76 were upregulated and 38 were downregulated in the male group compared to the female group (Figure 2B). A heat map showed that the expression profiles of these 114 differentially expressed piRNAs showed a strong correlation among samples in the same group (Figure 2B). These piRNAs were employed for target prediction, and 107 of 114 differentially expressed piRNAs were predicted to target 18,046 target genes.

### 2.5. Gene Ontology and Kyoto Encyclopedia of Genes and Genomes Pathway Enrichment Analyses

**Figure 3:**
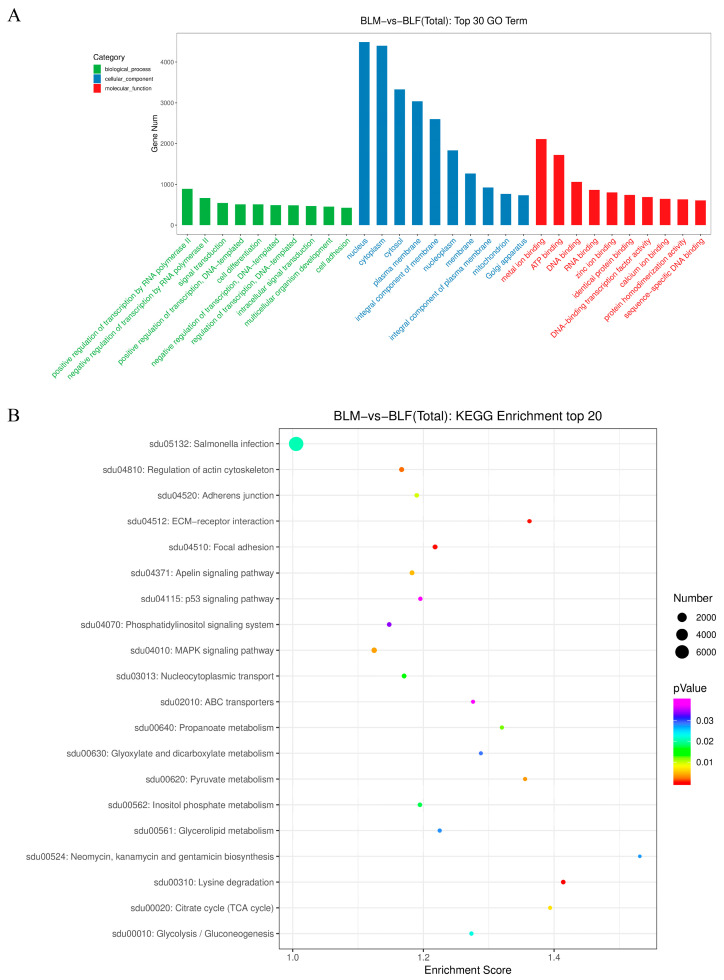
Gene Ontology terms and Kyoto Encyclopedia of Genes and Genomes pathways for target genes of differentially expressed piwi-interacting RNAs. (**A**) Bar chart of the top 30 significantly enriched Gene Ontology terms in three categories (biological process, cellular component, and molecular function) for target genes of differentially expressed piwi-interacting RNAs in the male and female groups. The *x*-axis shows the terms, and the *y*-axis shows the number of the target genes corresponding to the terms. (**B**) Bubble chart of the top 20 significantly enriched Kyoto Encyclopedia of Genes and Genomes pathways for target genes of differentially expressed piwi-interacting RNAs in the male and female groups. The *x*-axis shows the enrichment score of each Kyoto Encyclopedia of Genes and Genomes pathway, and the *y*-axis represents the name and ID of the Kyoto Encyclopedia of Genes and Genomes pathway. Each dot corresponds to a pathway. The color of the dot represents the *p*-value. A redder color indicates a smaller *p*-value, while purple indicates a larger *p*-value. The size of the dot represents the number of target genes involved in the pathway, where a larger size indicates more target genes.

The 18,046 target genes were evaluated by Gene Ontology (GO) enrichment analysis and Kyoto Encyclopedia of Genes and Genomes (KEGG) pathway enrichment analyses. The target genes were associated with 7633 GO terms and 155 KEGG pathways. The top 30 GO terms (including the top 10 GO terms in the biological process, cellular component, and molecular function categories) included cell differentiation and sex-related terms (Figure 3A). The KEGG enrichment analysis revealed that most target genes were enriched in the top 20 KEGG pathways, and the top five KEGG pathways included lysine degradation, focal adhesion, extracellular matrix (ECM)–receptor interaction, regulation of actin cytoskeleton, and pyruvate metabolism (Figure 3B). Based on these functional enrichment analyses, the target genes of 79 differentially expressed piRNAs were related to sex development, sex differentiation, sex determination, sex-related hormones biosynthetic process, and response to sex-related hormones. The 79 sex-related piRNAs were used for further analyses.

### 2.6. Real-Time Quantitative Polymerase Chain Reaction Verification of Signature Piwi-Interacting RNAs with Differential Expression between Male and Female Greater Amberjack

**Figure 4:**
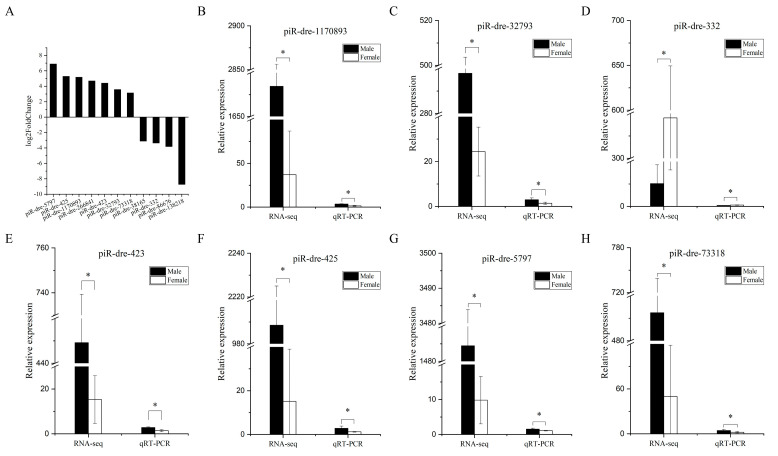
Differential expression of 11 candidate signature piwi-interacting RNAs between male and female greater amberjack based on transcripts per million values from small RNA sequencing, and comparisons of piwi-interacting RNA expression patterns from RNA-sequencing and real-time quantitative polymerase chain reaction analyses. (**A**) Differential expression of 11 candidate signature piRNAs between male and female groups based on transcripts per million values from small RNA sequencing. The *x*-axis shows the name of each piwi-interacting RNA, and the *y*-axis represents the log_2_ fold change value of the piwi-interacting RNA. (**B**–**H**) Comparisons of seven piwi-interacting RNA expression patterns in serum exosomes from RNA-sequencing and real-time quantitative polymerase chain reaction analyses. The *x*-axis represents RNA-sequencing and real-time quantitative polymerase chain reaction pairs; the *y*-axis represents the relative expression levels of piwi-interacting RNAs. The black column (left column) represents the relative expression of piwi-interacting RNAs in the male group, and the white column (right column) represents the relative expression of piwi-interacting RNAs in the female group. Data are presented as the mean ± SD, n = 3. *, *p* < 0.05. (**B**) piR-dre-1170893. (**C**) piR-dre-32793. (**D**) piR-dre-332. (**E**) piR-dre-423. (**F**) piR-dre-425. (**G**) piR-dre-5797. (**H**) piR-dre-73318. piR-dre-32793.

**Figure 5:**
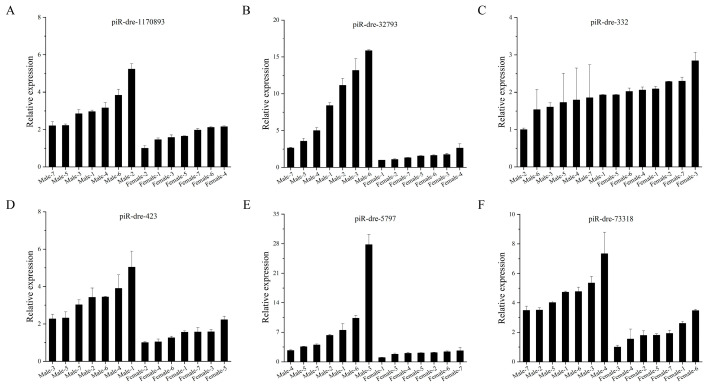
Real-time quantitative polymerase chain reaction verification of the expression of six piwi-interacting RNAs in serum exosomes from seven male and seven female greater amberjack. The *x*-axis represents each fish from the male and female group, and the *y*-axis represents the relative expression levels of piwi-interacting RNAs in each fish. Data are presented as the mean ± SD, n = 3. (**A**) piR-dre-1170893. (**B**) piR-dre-32793. (**C**) piR-dre-332. (**D**) piR-dre-423. (**E**) piR-dre-5797. (**F**) piR-dre-73318.

**Figure 6:**
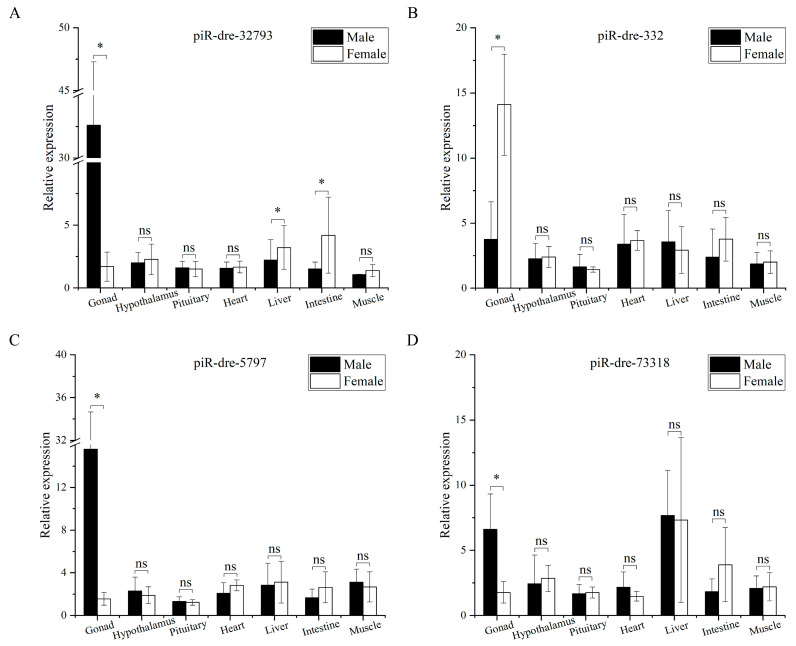
Real-time quantitative polymerase chain reaction verification of four piwi-interacting RNA expression patterns in seven different tissues from three male and three female greater amberjacks. The *x*-axis represents different tissues (gonad, hypothalamus, pituitary, heart, liver, intestine, and muscle) from the male and female group, and the *y*-axis represents the relative expression of piwi-interacting RNAs in the male and female group. The black column (left column) represents the relative expression of piRNAs in the male group, and the white column (right column) represents the relative expression of piwi-interacting RNAs in the female group. Data are presented as the mean ± SD, n = 3. *, *p* < 0.05. ns, *p* > 0.05. (**A**) piR-dre-32793. (**B**) piR-dre-332. (**C**) piR-dre-5797. (**D**) piR-dre-73318.

Among 79 sex-related piRNAs, there were 25 sex-inclined piRNAs in all three samples from the male group with higher or lower expression levels than from those in the female group according to small RNA sequencing. Furthermore, eleven differentially expressed piRNAs (seven up-regulated piRNAs and four down-regulated piRNAs) in serum exosomes were chosen as candidate signature piRNAs for verification by real-time quantitative polymerase chain reaction (qPCR), with TPM expression levels exceeding 100 in at least one of six sequenced samples (Figure 4A). Six piRNAs of eleven candidate piRNAs were significantly up-regulated in male fish compared with levels in female fish, while levels of one piRNA were significantly lower in male fish than in female fish (*p* < 0.05); the results for these seven piRNAs were consistent with the piRNA profiles obtained by small RNA sequencing (Figure 4). Furthermore, additional serum exosomal samples (seven male fish versus seven female fish) were employed for expanded testing by qPCR. In this analysis, results for six piRNAs were consistent with those of small RNA sequencing; expression levels of five were significantly higher in male fish than in female fish, while levels of one piRNA showed the opposite pattern (*p* < 0.05) (Figure 5). The highest relative expression of piR-dre-1170893, piR-dre-32793, piR-dre-423, piR-dre-5797, and piR-dre-73318 in seven female fish were 2.151435199, 2.64589873, 2.227002293, 2.64288962, and 2.610600076, respectively, and that of piR-dre-332 in seven male fish was 1.929653748 (Figure 5). Furthermore, results for four piRNAs (piR-dre-32793, piR-dre-332, piR-dre-5797, and piR-dre-73318) among the six verified piRNAs in gonadal samples from same individuals were consistent with results for serum exosomal samples. Levels of three piRNAs (piR-dre-32793, piR-dre-5797, and piR-dre-73318) were significantly higher in male fish than female fish, while levels of only piR-dre-332 showed the opposite pattern (*p* < 0.05) (Figure 6). Therefore, four signature piRNAs (piR-dre-32793, piR-dre-332, piR-dre-5797, and piR-dre-73318) were identified as promising molecular markers for sex determination in greater amberjack. Based on the relative expression of four marker piRNAs in the serum exosomes, the highest relative expression of piR-dre-32793, piR-dre-5797, and piR-dre-73318 in seven female fish, and that of piR-dre-332 in seven male fish can be used as the standard for sex determination of greater amberjack. The method of sex identification can ascertain the sex of greater amberjack by blood collection from the living body, without sacrificing fish.

### 2.7. Expression of Sex-Inclined Piwi-Interacting RNAs in Other Tissues

The four candidate piRNAs were evaluated by qPCR in six other tissue types, including the hypothalamus, pituitary, heart, liver, intestine, and muscle (three male fish versus three female fish). The expression levels of the four piRNAs in six tissue samples from male and female fish were not consistent with the results obtained for the gonads or serum exosomes (Figure 6). There were no significant expression differences in three piRNAs (piR-dre-332, piR-dre-5797, and piR-dre-73318) in six tissue samples between male and female fish (*p* > 0.05). The expression levels of piR-dre-32793 did not differ significantly between sexes in hypothalamus, pituitary, heart, and muscle samples (*p* > 0.05) but were significantly lower in the liver and intestine in male fish than in female fish (*p* < 0.05).

### 2.8. Piwi-Interacting RNAs–Target Interaction Network Analysis

**Figure 7:**
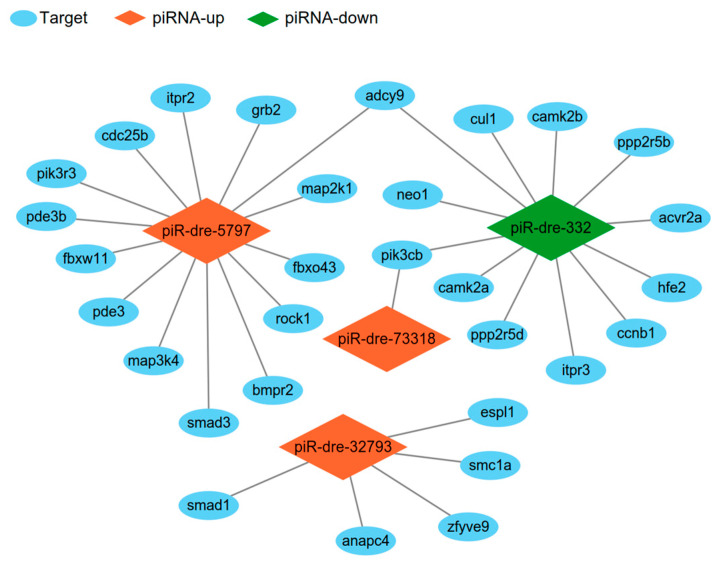
Piwi-interacting RNAs–target interaction network analysis. The orange rhombus represents up-regulated piwi-interacting RNAs in the male fish compared to levels in female fish, while the green rhombus represents down-regulated piwi-interacting RNAs in male fish. The blue ellipse represents target genes enriched in sex-related pathways.

Four signature piRNAs (piR-dre-32793, piR-dre-332, piR-dre-5797, and piR-dre-73318) were used to construct piRNA–target interaction networks. The sex-related interaction networks included 32 piRNA–target genes pairs (Figure 7), and these were enriched in four sex-related pathways, including oocyte meiosis, transforming growth factor-beta (TGF-β) signaling pathway, progesterone-mediated oocyte maturation, and gonadotropin releasing hormone (GnRH) signaling pathway. In this interaction network, piR-dre-32793, piR-dre-332, piR-dre-5797, and piR-dre-73318 all targeted more than one gene and were involved in multiple pathways. Additionally, *adcy9* (adenylate cyclase 9) and *pik3cb* (phosphatidylinositol-4,5-bisphosphate 3-kinase catalytic subunit beta) were targeted by more than one piRNA. PiR-dre-332 was a hub in these networks. In addition, both piR-dre-332 and piR-dre-5797 targeted *adcy9*, and both piR-dre-332 and piR-dre-73318 targeted *pik3cb*.

## 3. Discussion

The mechanisms underlying sex determination in fish are complex and diverse, involving various regulatory factors [36,37]. Studies have increasingly focused on the role of the epigenetic regulation of gene expression in reproductive endocrinology. As non-coding RNA (ncRNA), piRNAs play an important regulatory role in the reproductive process, especially in gonadal tissues, and could be biomarkers for sex determination [30]. In this study, sex-inclined piRNAs in serum exosomes of greater amberjack were identified as molecular markers for sex determination. The method of sex identification can ascertain the sex of greater amberjack by measuring the relative expression of marker piRNAs in serum exosomes by blood collection from the living body, without sacrificing fish. These markers provide a tool for the accurate identification of the sex of greater amberjack and improve our understanding of the molecular mechanism underlying piRNA-mediated sex differentiation.

Differentially expressed piRNAs were identified in the serum exosomes of greater amberjack, as in the testis or/and ovary of other teleosts, including zebrafish (*Danio rerio*) [38], common carp (*Cyprinus carpio*) [39], Nile tilapia (*Oreochromis niloticus*) [40,41], Atlantic salmon (*Salmon salar* L.) [42], turbot (*Scophthalmus maximus*) [43,44], sharpsnout seabream (*Diplodus puntazzo*) [45], Japanese flounder (*Paralichthys olivaceus*) [46,47,48], and *C. semilaevis* [34,35,49]. In these studies, the number of known piRNAs ranged from 13 in the gonads of *D. puntazzo* [45] to 296,775 piRNAs in the testis of *O. niloticus* [41]. In this study, more than 1000 known piRNAs were identified, 114 of which were differentially expressed piRNAs, supporting the validity of the sequencing results.

In this study, four signature piRNAs (piR-dre-32793, piR-dre-332, piR-dre-5797, and piR-dre-73318) were differentially expressed in serum exosomes and gonads between male and female greater amberjack at one year of age. Furthermore, the expression patterns of these four signature piRNAs (piR-dre-32793, piR-dre-332, piR-dre-5797, and piR-dre-73318) in serum exosomes and gonads were not consistent with those in six other tissues (hypothalamus, pituitary, heart, liver, intestine, and muscle), showing that these piRNAs have tissue-specific sex-inclined expression; furthermore, expression patterns in the serum were more similar to those in the gonads than to those in other tissue types. Tissue-specific expression of piRNAs has also been found in other fish, including *S. salar* L. [42], *S. maximus* [43,44], and *P. olivaceus* [47,48]. *Piwil1* and *piwil2* were specifically highly expressed in the testis and ovary but were expressed at low or negligible levels in other tissues, including the brain, fin, skin, muscle, liver, vertebrae, intestine, eye, heart, spleen, kidney, and gill [42,43,44,47,48]. Consistent with these previous results, four signature piRNAs identified in this study were highly expressed in the gonads and expressed at low levels in other tissues, including the hypothalamus, pituitary, heart, liver, intestine and muscle, further verifying the tissue-specific expression of piRNAs.

Furthermore, the four signature piRNAs and their target genes were related to in utero embryonic development, oocyte meiosis, the TGF-β signaling pathway, progesterone-mediated oocyte maturation, and the GnRH signaling pathway. In the hypothalamus–pituitary–gonad axis in fish, GnRH secreted from the hypothalamus regulates gonadotropin (Gn) secretion from the pituitary, thus regulating sex hormone secretion and synthesis from the gonads [50,51,52,53]. In addition, sex hormone-binding globulin (SHBG) in the blood binds to and transports hormones, including GnRH, Gn, and sex hormones, to target organs, including gonads [54,55,56]. The hypothalamus and pituitary are upstream of the hypothalamus–pituitary–gonad axis, and their piRNAs may exhibit asynchronous expression with that of piRNAs in the gonads and serum. Gonads are the direct target organs of sex hormones transported by blood, and most piRNAs that have been identified as sex-inclined are derived from and act on gonads [30], which may explain the similar expression patterns between serum exosomes and gonads in greater amberjack.

The four signature piRNAs and their target genes were involved in sex-related pathways. Oocyte meiosis and progesterone-mediated oocyte maturation were female-inclined pathways. Furthermore, the four piRNAs and most of their target genes (*smc1a*, *espl1*, *anapc4*, *ccnb1*, *ppp2r5d*, *adcy9*, *ppp2r5b*, *pik3cb*, *itpr3*, *cul1*, *camk2a*, *camk2b*, *cdc25b*, *pik3r3*, *fbxo43*, *itpr2*, *map2k1*, *pde3b*, and *fbxw11*) were involved in oocyte meiosis or/and progesterone-mediated oocyte maturation, suggesting that the piRNAs are male-inclined and their target genes are generally female-inclined.

*Adcy9* was a target gene of piR-dre-332 and piR-dre-5797. *Adcy9*, which encodes an adenylyl cyclase [57,58,59,60], can regulate the metabolism associated with the estrogen receptor pathway and is a sex-specific gene in patients with coronary artery disease [61,62]. In addition, *adcy9* is a female-inclined gene that is regulated by an estrogen-related receptor (ERR) to modulate ovarian development in the giant freshwater prawn (*Macrobrachium rosenbergii*) [63]. *Adcy9* may be simultaneously modulated by the down-regulation of piR-dre-332 and up-regulation of piR-dre-5797 to participate in progesterone-mediated oocyte maturation; this will be further investigated in subsequent studies.

*Pik3cb* was a target gene of piR-dre-332 and piR-dre-73318. *Pik3cb* encodes p110β catalytic subunits of PI3K, a significant regulator of cell growth, differentiation, survival, proliferation, migration, and metabolism [64,65,66,67,68]. *Pik3cb* is highly expressed in obese individuals during pregnancy and can increase insulin sensitivity in offspring, which is beneficial for the metabolic health of offspring after weaning [69]. In addition, *pik3cb* showed low expression in the chronic sleep restriction group, and it was related to the prolactin (PRL) signaling pathway; the expression of *pik3cb* could be impacted by chronic sleep restriction to interrupt the *Pi3k/Akt* signaling pathways, further inhibiting spermatogenesis [70].

The four piRNAs (piR-dre-32793, piR-dre-332, piR-dre-5797, and piR-dre-73318) were not only significantly differentially expressed between male and female greater amberjack but also targeted many sex-related target genes, suggesting that the four piRNAs are very promising biomarkers for sex determination. However, it is still unclear how these piRNAs interact with their target genes to regulate gonadal differentiation and development, and further research should focus on the regulatory mechanisms of these piRNA–target pairs.

## 4. Materials and Methods

### 4.1. Ethics Statement

The greater amberjack specimens were collected from Zhangzhou, Fujian, and used as serum donors. The greater amberjack experiments were approved by the academic ethics committee of Guangdong Ocean University and were carried out under the relevant guidelines of the Laboratory Animal Center of Guangdong Ocean University.

### 4.2. Sampling, Histological Analysis and Isolation of Serum Exosomes from Greater Amberjack

Three male (male group) and three female (female group) one-year-old greater amberjack were evaluated. The appearance of males was similar to that of females. The gonadal tissues of each fish were collected and placed in a centrifuge tube with 4% paraformaldehyde. Blood samples were collected from each fish and added to a centrifuge tube on ice for centrifugation.

To accurately identify the sex of greater amberjack, the gonadal samples were used to make paraffin sections for hematoxylin-eosin staining [2,71]. The gonadal samples were firstly fixed by paraformaldehyde solution overnight, followed by gradient dehydration with ethanol. The dehydrated samples were treated with anhydrous xylene-ethanol mixtures and anhydrous xylene at room temperature. The transparent samples were dipped in xylene–paraffin mixtures and paraffin, and the gonad blocks embedded in paraffin were sectioned (width, 5 μm). Paraffin was removed from slides containing sections by dipping in anhydrous xylene and anhydrous xylene–ethanol mixtures. The slides with xylene were washed with gradient ethanol and distilled water and were stained with hematoxylin for 5 min. The stained slides were washed in running water and gradient ethanol, followed by staining with acid-based eosin. Slides were dehydrated with gradient ethanol, dipped in anhydrous xylene and anhydrous xylene-ethanol mixtures, and finally mounted with sealing liquid. Images of tissue slices were captured using a Nikon ECLIPSE Ni light microscope (Nikon, Tokyo, Japan) with the Nikon DS-Ri2 camera.

Blood samples were maintained on ice for several hours and centrifuged at 3500× *g* for 10 min (4 °C). Serum samples of approximately 1 mL were isolated. Subsequently, exosomes from the serum samples were isolated following the instructions provided with Total Exosome Isolation Reagent (ThermoFisher Scientific, Waltham, MA, USA). The mixtures of serum samples from each fish and Total Exosome Isolation Reagent (2:1) were placed overnight at 4 °C and then centrifuged at 10,000× *g* for 1 h (4 °C). The supernatant was discarded and the centrifuge tube was inverted on filter paper for 2 min. The isolated exosomes were resuspended in 50 μL of phosphate-buffered saline (PBS) and stored at −20 °C for further identification and RNA extraction.

### 4.3. Characterization of Serum Exosomes of Greater Amberjack

#### 4.3.1. Transmission Electron Microscopy

For TEM, 5 μL of each exosome sample with PBS was fixed to a copper mesh at room temperature for 5 min. PBS around the exosomes was absorbed by filter paper [72]. The exosomes were dyed with saturated uranyl acetate solution for 1 min on the copper mesh and then the solution around the exosomes was absorbed by filter paper [73]. Enzyme-free water (ddH_2_O) was dripped on the exosomes on the copper mesh at room temperature for 5 min and absorbed by filter paper, and this process was repeated once. After drying at room temperature, the exosomes were observed under a transmission electron microscope (Tecnai G2 Spirit; FEI, Hillsboro, OR, USA). Images of exosomes were captured using a digital camera (Sony, Tokyo, Japan) [34].

#### 4.3.2. Nanoparticle Tracking Analysis

For a NTA, 20 μL of exosomes was diluted with PBS to 1000 μL and mixed homogeneously with a vortex mixer. The Malvin Nanosight NS300 (Malvern Instruments, Westborough, MA, USA) was used for the quantification and size analysis of diluted exosomes, repeated three times for 30 s each time [34,49]. Data were analyzed using NTA 2.3 analysis software [34,49].

#### 4.3.3. Western Blotting Analysis

For western blotting (WB), 100 μL of exosomes was lysed with 200 μL of RIPA Lysis Buffer with protease inhibitor (Roche, Basel, Switzerland) and phosphatase inhibitors (Roche) at 4 °C for 15 min. The lysed exosomes were centrifuged at 12,000× *g* at 4 °C for 5 min to obtain the supernatant [74]. The supernatant was subjected to sodium dodecyl sulfate-polyacrylamide gel electrophoresis (SDS-PAGE) to obtain the exosomal proteins, which were transferred to PVDF membranes (Millipore Corp., Bedford, MA, USA) [75]. At room temperature, the PVDF membranes were sealed with 5% BSA for 2 h and were washed three times with TBST on a shaker for 5 min each time. Then, the membranes were incubated at 4 °C overnight in an antibody incubation bag with specific primary antibodies, including anti-rabbit CD63 antibody (ab134045, ABCAM, Cambridge, UK), anti-rabbit CD9 antibody (ab236630, ABCAM), and anti-rabbit HSP70 antibody (10995-1-ap, Proteintech, Rosemont, IL, USA) [75,76,77]. After washing three times with TBST for 5 min each time, the membranes were incubated with a horseradish peroxidase (HRP)-labelled AffiniPure Goat Anti-Rabbit IgG (H+L) (111-035-003, 1:5000, Jackson ImmunoResearch) at room temperature for 2 h [76]. Subsequently, the membranes were washed five times with TBST for 15 min each time. The membranes were reacted with chemiluminescence reagents for 2 min and visualized using a chemiluminescence imaging system (BLT, GV 6000M2). Gray scale values were determined using IPP6.0.

### 4.4. Small RNA Library Construction, Sequencing, and Bioinformatics Analysis

Total RNAs were isolated from serum exosomes using AG RNAex Pro Reagent (AG21102; Accurate Biology, Changsha, Hunan, China) and the quality and concentration were evaluated using the Agilent Bioanalyzer (Santa Clara, CA, USA). High-quality RNA from three female (female group) and three male greater amberjack (male group) were sequenced separately. Six small RNA libraries were constructed and used for Illumina HiSeq sequencing (OE biotech Co., Ltd., Shanghai, China).

Raw reads (original sequencing data, also called raw data) were obtained by base calling (a sequencing process of the inference of DNA sequences from physical signals) and were used for further quality control. To obtain clean reads, reads with adapter sequences were filtered using cutadapt (version 1.14) [78] and the reads with 15–41 nt were retained. Additionally, reads with Q20 < 80% were removed using fastx_toolkit (version 0.0.13) [79], and reads with N bases were filtered using NGSQCToolkit (version 2.3.3) [80]. The length distribution of the clean sequences in the reference genome was determined. To classify and annotate the small RNAs, clean reads were compared against the Rfam database [81], GenBank (http://www.ncbi.nlm.nih.gov/genbank/, accessed on 23 November 2021) databases, miRBase database [82,83], and piRBase database [84]. The distribution of small RNAs from each sample was analyzed and classified. Reads that were 18–34 nt and aligned to the piRBase database were considered known piRNAs. The expression patterns of known piRNAs in six samples were calculated using the transcripts per million (TPM) method as follows: TPM = N/M*10^6^ [85], where N refers to clean reads aligned to each piRNA and M represents clean reads aligned to the total piRNAs of each sample. Differentially expressed piRNAs between the male group (three male fish) and female group (three female fish) were identified with *p* < 0.05 and |log_2_ FC| > 2 as thresholds, and the *p*-value for each piRNA was calculated using the DEG algorithm [86] in the DESeq R package with biological replicates. The expression levels of differentially expressed piRNAs were used to evaluate correlations among samples, and a heat map was generated for visualization. The target genes of differentially expressed piRNAs were predicted using miRanda [87] with the following parameters: S ≥ 150, ΔG ≤ −30 kcal/mol, and strict 5′ seed pairing. The predicted target genes were evaluated by a GO enrichment analysis and KEGG pathway enrichment analysis using the Stats R-package based on the hypergeometric distribution.

Differentially expressed exosome piRNAs in the serum were chosen as candidate signature piRNAs with the following standards. (1) Differentially expressed piRNAs are sex-related. (2) The expression levels of each piRNA in three samples from the male group were higher or lower than those of three samples from female group. (3) In the six samples, the piRNA expression level of at least one sample was higher than 100. The log_2_ FC of the candidate signature piRNA was evaluated. The expression levels of the candidate signature piRNAs were assayed by qPCR, using oni-let-7a as an internal reference.

### 4.5. Real-Time Quantitative Polymerase Chain Reaction of Differentially Expressed Piwi-Interacting RNAs

Candidate signature piRNAs were selected for qPCR with serum exosomal samples (three male fish versus three female fish) for validation. When qPCR results were consistent with the sequencing results, piRNAs were selected for qPCR analysis of additional serum exosomal samples (seven male fish versus seven female fish, not used for sequencing). Those piRNAs with 100% consistency across analyses were selected for qPCR of gonadal samples (three male fish versus three female fish), an important organ showing primary sexual characteristics. Finally, piRNAs whose expression patterns were consistent with the serum piRNA sequencing results were selected for qPCR analyses of other tissues, including the hypothalamus, pituitary, heart, liver, intestine, and muscle samples (three male fish versus three female fish), i.e., organs without primary sexual characteristics, to evaluate tissue specificity. The verified piRNAs were considered signature piRNAs.

Total RNAs from serum exosomes were isolated using AG RNAex Pro Reagent (AG21102, Accurate Biology, Changsha, Hunan, China), while total RNAs from seven other tissue types (gonad, hypothalamus, pituitary, heart, liver, intestine, and muscle) were isolated using TRIzol Reagent (Invitrogen, Waltham, MA, USA). Total RNAs from the serum exosomes (10 ng) and other tissues (400 ng) from the male group and female group were used for reverse transcription. The specific stem-loop primers for reverse transcription and specific quantitative primer pairs of the candidate signature piRNAs and the internal reference (oni-let-7a) were designed using miRNA Design (Version 1.01) and Primer3 (Version 0.4.0) (shown in Supplementary Table S1). According to the manufacturer’s recommendations, the cDNAs were synthesized with specific stem-loop primers for the candidate signature piRNAs and the internal reference, respectively, using the miRNA 1st Strand cDNA Synthesis Kit (by stem-loop) (MR101-01, Vazyme, Nanjing, China) and a thermal cycler (Thermo Fisher Scientific). According to the manufacturer’s protocol, the cDNAs were used for qPCR with specific primer pairs for the candidate signature piRNAs and the internal reference using miRNA Universal SYBR qPCR Master Mix (MQ101-02, Vazyme) in 384-well reaction plates and the Roche LightCycler 480-II instrument. The qPCR program was set as follows: 1 cycle of 5 min at 95 °C, 40 cycles of 30 s at 94 °C, 20 s at 58 °C, and 20 s at 72 °C, 1 cycle of 10 s at 95 °C and 60 s at 65 °C, and 1 cycle of 30 s at 37 °C. Each sample was run in triplicate, and the relative expression levels of piRNAs were calculated with the 2^−ΔΔCt^ method [88]. The relative expression levels are shown as mean ± SD.

### 4.6. Piwi-Interacting RNAs–Target Interaction Network Analysis

Based on the results of qPCR validation and the KEGG pathway enrichment analysis, the validated signature piRNAs and their predicted targets were selected to construct a piRNA–target interaction network. The piRNA–mRNA pairs expressed in the serum of greater amberjack were selected from the sex-related pathways, and their interaction networks were constructed using Cytoscape (Version 3.5.1) (http://www.cytoscape.org/, accessed on 30 September 2022).

### 4.7. Statistical Analysis

Statistical analyses were performed using SPSS 25.0. The independent samples t-test was used to evaluate differences between the male group and female group. Parameters, including body length, body weight, and qPCR results, are shown as the mean ± SD. A *p*-value < 0.05 was recognized as a significant difference.

## 5. Conclusions

In this study, piRNA profiles of serum exosomes from greater amberjack were characterized to screen sex-inclined piRNAs as molecular markers for sex determination in the species. Four piRNAs that were differentially expressed in the serum exosomes and gonads between male and female greater amberjack were identified as signature piRNAs for sex determination, and their sex-related target genes were enriched in sex-related pathways, including oocyte meiosis, the TGF-β signaling pathway, progesterone-mediated oocyte maturation, and the GnRH signaling pathway. This work provides biomarkers for the identification of the sex of greater amberjack based on piRNAs from serum exosomes and provides insight into the interactions between piRNAs and sex-related target genes in the regulation of sex development and differentiation in greater amberjack.

## Data Availability

Raw data have been uploaded and are available on the NCBI website (Accession No. PRJNA896259).

## Supplementary Materials

The following supporting information can be downloaded at: https://www.mdpi.com/article/10.3390/ijms24043438/s1.
