# Supplementary material for: Sex-Inclined Piwi-Interacting RNAs in Serum Exosomes for Sex Determination in the Greater Amberjack (Seriola dumerili)
Source: Int J Mol Sci. 2023 Feb 8;24(4):3438. doi: 10.3390/ijms24043438 (PMC9962539; doi:10.3390/ijms24043438)
Supplement: Supplementary file 1 [file ijms-24-03438-s001.zip › ijms-2137357-supplementary.pdf]

Table S1: The specific stem-loop primers for reverse transcription and specific quantitative primer pairs of the candidate signature piRNAs and the internal reference (oni-let-7a). These primers were designed using miRNA Design (Version 1.01) and Primer3 (Version 0.4.0).

ID	Primer name	Sequence（5' to 3'）	Length
01	piR-dre-1170893-RT	GTCGTATCCAGTGCAGGGTCCGAGGTATTCGC ACTGGATACGACTTCCCA	50
02	piR-dre-1170893-F	AAGGTGTTCGACTCCCGGTA	20
03	piR-dre-1170893-R	AGTGCAGGGTCCGAGGTATT	20
04	piR-dre-138218-RT	GTCGTATCCAGTGCAGGGTCCGAGGTATTCGC ACTGGATACGACTGGGAA	50
05	piR-dre-138218-F	GACAGTTCGATGTCGGCTC	19
06	piR-dre-138218-R	AGTGCAGGGTCCGAGGTATT	20
07	piR-dre-266841-RT	GTCGTATCCAGTGCAGGGTCCGAGGTATTCGC ACTGGATACGACATGGGA	50
08	piR-dre-266841p-F	AGCTTCGATGTCGGCTCT	18
09	piR-dre-266841-R	AGTGCAGGGTCCGAGGTATT	20
10	piR-dre-32793-RT	GTCGTATCCAGTGCAGGGTCCGAGGTATTCGC ACTGGATACGACCGGCGG	50
11	piR-dre-32793-F	TGAGGTCCTCGGATCGGCC	19
12	piR-dre-32793-R	AGTGCAGGGTCCGAGGTATT	20
13	piR-dre-332-RT	GTCGTATCCAGTGCAGGGTCCGAGGTATTCGC ACTGGATACGACTCCCAT	50
14	piR-dre-332-F	GGGTTCGACTCCCGGT	16
15	piR-dre-332-R	AGTGCAGGGTCCGAGGTATT	20
16	piR-dre-38165-RT	GTCGTATCCAGTGCAGGGTCCGAGGTATTCGC ACTGGATACGACACCAGG	50
17	piR-dre-38165-F	CGGGTCTAGCGGTTAGGATT	20
18	piR-dre-38165-R	AGTGCAGGGTCCGAGGTATT	20
19	piR-dre-423-RT	GTCGTATCCAGTGCAGGGTCCGAGGTATTCGC ACTGGATACGACTAAGCT	50
20	piR-dre-423-F	CTGGGAATACCAGGTGCTGTA	21
21	piR-dre-423-R	AGTGCAGGGTCCGAGGTATT	20
22	piR-dre-425-RT	GTCGTATCCAGTGCAGGGTCCGAGGTATTCGC ACTGGATACGACTAAGCT	50
23	piR-dre-425-F	CCTGGGAATACCAGGTGCTGTA	22
24	piR-dre-425-R	AGTGCAGGGTCCGAGGTATT	20
25	piR-dre-5797-RT	GTCGTATCCAGTGCAGGGTCCGAGGTATTCGC ACTGGATACGACCCCGTC	50
26	piR-dre-5797-F	GAGACCGGGGTTCGATT	17
27	piR-dre-5797-R	AGTGCAGGGTCCGAGGTATT	20
28	piR-dre-73318-RT	GTCGTATCCAGTGCAGGGTCCGAGGTATTCGC ACTGGATACGACACCCTG	50
29	piR-dre-73318-F	AGTAGACGACCTGATTCTGGGT	22

30	piR-dre-73318-R	AGTGCAGGGTCCGAGGTATT	20
31	piR-dre-86676-RT	GTCGTATCCAGTGCAGGGTCCGAGGTATTCGC ACTGGATACGACGGACGA	50
32	piR-dre-86676-F	TGAGCACGCCCGATC	15
33	piR-dre-86676-R	AGTGCAGGGTCCGAGGTATT	20
34	oni-let-7a-RT	GTCGTATCCAGTGCAGGGTCCGAGGTATTCGC ACTGGATACGACAACTAT	50
35	oni-let-7a-F	GCGCGTGAGGTAGTAGGTTGT	21
36	oni-let-7a-R	AGTGCAGGGTCCGAGGTATT	20

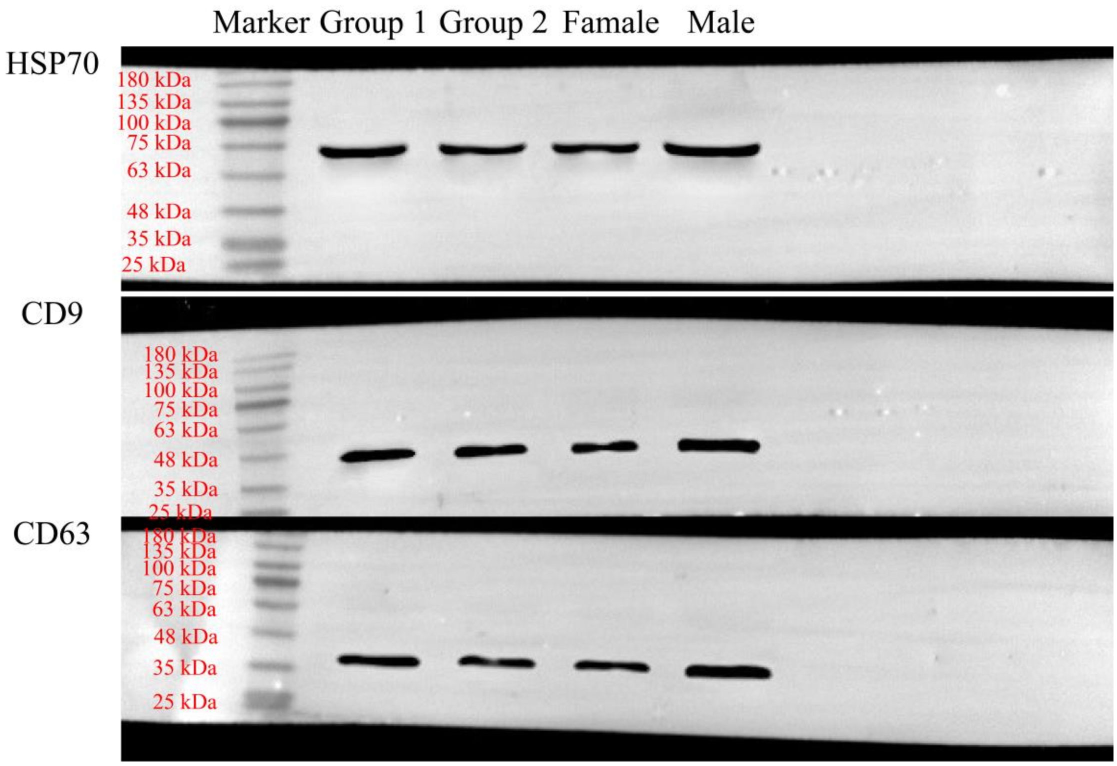

Figure S1. The full gel pictures of western blotting for the detection of HSP70, CD9, and CD63. Group 1 and group 2 were the results of exosome identification of samples from other experiments. Female and male were the results of exosome identification of samples in this study.
